# Synthesis of Carboxymethyl Dextran-Coated Gold Nanoparticles as Stable and Storable Optical Labels for Ultrasensitive Plasmonic Nanoparticle-Linked Sorbent Assay

**DOI:** 10.3390/s25237156

**Published:** 2025-11-24

**Authors:** Novi Asri Sitinjak, Chien-Wei Huang, Tsung-Yi Yang, Lai-Kwan Chau, Chih-Hsien Wang

**Affiliations:** Department of Chemistry and Biochemistry, Center for Nano Bio-Detection, National Chung Cheng University, Chiayi 621301, Taiwan; novi.sitinjak@gmail.com (N.A.S.); chienwei880917@gmail.com (C.-W.H.); laurence880721laurence@gmail.com (T.-Y.Y.)

**Keywords:** carboxymethyl dextran, carboxymethyl dextran-coated gold nanoparticles, biosensor, fiber optic nanogold-linked sorbent assay

## Abstract

Gold nanoparticles (AuNPs) are widely employed in biosensors; however, conventional synthesis methods require additional surface modification to confer colloidal stability and bioconjugation capability. Here, we report a facile strategy to synthesize carboxymethyl dextran (CMD)-coated AuNPs (AuNP@CMD) that simultaneously serve as a plasmonic label, a stabilizing agent, and a functional scaffold. The CMD was prepared directly via partial carboxymethylation of dextran in a one-pot reduction of HAuCl_4_, enabling the synthesis of AuNP@CMD with tunable particle sizes and excellent colloidal stability for at least one month at 4 °C. The CMD coating on AuNPs can prevent nanoparticle aggregation, suppress nonspecific adsorption, and introduce surface carboxyl groups for conjugation of bioprobes. Such characteristics are important to develop plasmonic nanoparticle-linked sorbent assays as an alternative to the conventional colorimetric enzyme-linked immunosorbent assay. When applied to a fiber-optic nanogold-linked sorbent assay, AuNP@CMD enabled ultrasensitive detection of a single-stranded DNA, achieving a detection limit at the femtomolar (fM) concentration level without nucleic acid amplification.

## 1. Introduction

Biosensors demand reliability, accessibility, and user-friendliness outside of controlled laboratory environments, which continue to pose challenges for the development of point-of-care testing (POCT). In recent years, gold nanoparticles (AuNPs) have been widely employed in biosensor applications. Due to the strong light extinction properties of AuNPs in their localized surface plasmon resonance (LSPR) band, they are often utilized as a colorimetric label for sandwich immunoassays [1,2,3,4,5,6,7] and become an alternative to the conventional colorimetric enzyme-linked immunosorbent assay (ELISA). In comparison with ELISA, these plasmonic nanoparticle-linked sorbent assays keep the basic workflow in ELISA but do not need an enzyme to amplify the colorimetric signal, and thus offer significantly shorter analysis time and less laborious procedures.

The most common approach for synthesizing AuNPs is the citrate reduction method [8]. However, this method typically requires additional surface modification steps to render AuNPs suitable for biosensing [9,10,11,12,13]. Furthermore, when applied in biological systems, AuNPs coated by non-biocompatible molecules may be harmful to those systems. In this regard, AuNPs coated by a biocompatible biopolymer layer are obviously an option. Under this consideration, development of biocompatible polysaccharide-capped AuNPs, especially dextran-capped AuNPs, has been reported [14,15,16,17,18,19,20,21,22]. However, such dextran-capped AuNPs still require additional steps for biomolecule immobilization [19,20]. To streamline the process, we introduced a one-pot reaction to prepare AuNPs having a protective layer that simultaneously provides reactive sites for bioconjugation and an antifouling surface to minimize nonspecific adsorption, as well as furnishing good dispersibility in aqueous solvents. Specifically, carboxyl functional groups were incorporated via Williamson ether synthesis [23] to generate partially carboxymethylated dextran (CMD). This was subsequently used to prepare CMD-coated gold nanoparticles (AuNP@CMD), wherein dextran directly reduces HAuCl_4_ to AuNPs under alkaline conditions [14,15]. The CMD coating enhances colloidal stability, prevents aggregation, and preserves both plasmonic properties and bioconjugation capability of AuNPs over extended storage at room temperature [24]. Furthermore, based on the methods reported by Ma [25] and Wang [16], a simplified one-step synthesis was developed to prepare AuNP@CMD with tunable particle sizes, while omitting the conventional dialysis and freeze-drying steps.

We further applied our AuNP@CMD to one plasmonic nanoparticle-linked sorbent assay: Fiber-Optic Nanogold-LInked Sorbent Assay (FONLISA) [2]. In this system, a capture probe (CP) was immobilized on the fiber core surface, while a detection probe (DP) was conjugated to AuNPs in a solution to form a free AuNP@DP bioconjugate. Upon interactions of target molecules (T) in a sample with the immobilized CP and free AuNP@DP, sandwich nanocomplexes (AuNP@DP–T–CP) form on the fiber core surface, leading to a marked reduction in transmitted light intensity through the optical fiber. This configuration enabled high sensitivity detection, achieving limits of detection (LOD) at the femtomolar (fM) concentration levels [2,26,27,28,29]. In this study, we employ a target single-stranded DNA as a model analyte to evaluate the analytical performance of using AuNP@CMD as a plasmonic nanoprobe in plasmonic nanoparticle-linked sorbent assays. Hence, its hybridization with an immobilized ssDNA CP and free ssDNA DP-conjugated AuNPs will form nanocomplexes on the fiber core surface, achieving a LOD of 28.5 fM.

## 2. Materials and Methods

### 2.1. Materials and Reagents

Hydrogen tetrachloroaurate (III) trihydrate (HAuCl_4_·3H_2_O, 99.99%), sodium chloroacetate (ClCH_2_COONa), and 2-morpholinoethanesulfonic acid (MES) were bought from Alfa Aesar (Tewksburry, MA, USA). (3-Aminopropyl) triethoxysilance (APTES) was purchased from ACROS Organics (Geel, Belgium). Sodium hydroxide and Tween 20 were obtained from Showa (Tokyo, Japan). Dextran 70 (MW = ca. 70000) and carboxymethyl dextran sodium salt (MW = ca. 40000) were purchased from Tokyo Chemical Industry (Tokyo, Japan). Ethanol (EtOH, 99%) was acquired from HY Biocare Chemical (New York, NY, USA). 1-Ethyl-3-(3-dimethylaminopropyl)carbodiimide hydrochloride (EDC), acetic acid, N-hydroxysuccinimide (NHS), and acetone were obtained from Sigma-Aldrich (st. Louis, MO, USA). Tris(hydroxymethyl)aminomethane buffer (Tris-base), sodium dihydrogen phosphate anhydrous, and sodium phosphate dibasic anhydrous were procured from J.T.Baker (Phillipsburg, NJ, USA). Phosphate-buffered saline (PBS) tablets were acquired from Biovision (Zurich, Switzerland). Ultrapure water (18.2 MΩ cm) was produced by a Milli-Q water system (Millipore, Darmstadt, Germany) to prepare all aqueous solutions. Oligonucleotides were provided by GENEWIZ (Indianapolis, IN, USA), with their sequences are as following,

Target ssDNA (T): 5′-TGCCCAGGGC CTCACCACCA ACTTC-3′,
Detection probe (DP): 5′-AGGCCCTGGG CAAAAAAAAA AA-3′-NH_2_,
Capture probe (CP): NH_2_-5′-AAAAAAAAAA GAAGTTGGTG GTG-3′,
where the underlined part in DP and CP indicate that those oligonucleotides are complementary to T, and A_10_ is a spacer in DP and CP.

### 2.2. Synthesis and Characterization of CMD Powder and AuNP@CMD^P^

Carboxymethyl dextran powder (CMD^P^) was synthesized following the method described by Heinze et al. [30], with slight modifications. Briefly, 1 g of dextran (70 kDa, final conc. ≈ 0.714 mM) and 0.5030 g of sodium chloroacetate (final conc. ≈ 0.216 M) were dissolved in 10 mL of ultrapure water under stirring. Subsequently, 10 mL of 5 M NaOH (final conc. 2.5 M) was added, and the mixture was stirred at 60 °C for 90 min. The pH of the reaction mixture was then adjusted to neutral with 3.0 M HCl, followed by desalting through dialysis (MWCO 6–8 kDa, 2 L × 3 cycles) against ultrapure water. The dialyzed product was lyophilized to obtain white CMD^P^. The introduction of carboxyl groups was verified using Fourier-transform infrared (FT-IR) spectroscopy (VERTEX 70v, Bruker, Billerica, MA, USA).

For the synthesis of CMD^P^-coated AuNPs (AuNP@CMD^P^), 25 mL of 0.25 mM HAuCl_4_ solution was heated to boiling in a two-necked flask under stirring. Subsequently, 0.2 mL of CMD^P^ solution (35 mg) and 0.1 mL of 1 M NaOH were rapidly added. The mixture was stirred and maintained at boiling temperature for 25 min, then cooled to room temperature, yielding a wine-red AuNP@CMD^P^ colloidal solution.

### 2.3. One-Step Synthesis of AuNP@CMD with Different Particle Sizes

#### 2.3.1. Small-Size AuNP@CMD^5%^

A solution of CMD^5%^ was prepared by dissolving 50 mg of dextran (70 kDa) and 30 mg of sodium chloroacetate in 1 mL of 0.5 M NaOH (i.e., ~5% dextran), followed by heating at 60 °C for 20 h in a dry bath, and then allowed to cool gradually to room temperature. Next, 25 mL of a 0.275 mM HAuCl_4_ solution was placed in a two-neck flask and brought to a boil with stirring. Subsequently, 0.2 mL of the CMD^5%^ solution was rapidly introduced, and the mixture was maintained under stirring at boiling temperature for 30 min. The reaction mixture was then allowed to cool gradually to room temperature, yielding a wine-red AuNP@CMD^5%^ colloidal solution.

#### 2.3.2. Large-Size AuNP@CMD^1%^

The synthesis followed similar procedures to those described above, except that a solution of CMD^1%^ was prepared from a mixture of 10 mg dextran (70 kDa) and 5 mg sodium chloroacetate dissolved in 1 mL of 0.5 M NaOH (i.e., ~1% dextran) and heated at 60 °C for 20 h. After cooling, 0.2 mL of this CMD^1%^ solution was added rapidly to 25 mL of boiling HAuCl_4_ solution (0.275 mM) under stirring. The reaction was allowed to proceed for 30 min at boiling temperature before cooling to room temperature, affording a red-purple AuNP@CMD^1%^ colloidal solution.

The presence of carboxyl groups was confirmed using Fourier-transform infrared spectroscopy (VERTEX 70v, Bruker, Billerica, MA, USA). The synthesized CMD^5%^ and CMD^1%^ solutions were purified by removing unreacted sodium chloroacetate and NaOH using an Amicon Ultra Centrifugal Filter (50 kDa MWCO, Merck Millipore). Then, the solutions were washed with ultrapure water and subsequently freeze-dried.

### 2.4. Characterization of AuNP@CMD

UV–vis absorption spectra were obtained using a UV–vis spectrophotometer (JASCO V-570). The average particle diameter of AuNP@CMD was determined by transmission electron microscopy (TEM, JEOL JEM-2010, Tokyo, Japan). Zeta potential and dynamic light scattering (DLS) measurements were performed using a Malvern Zetasizer Nano ZS90 (Malvern, UK). Thermogravimetric analysis (TGA) was performed on a Simultaneous Thermal Analyzer (STA 6000, PerkinElmer, MA, USA). Samples were heated from 30 °C to 800 °C under a nitrogen atmosphere at a constant heating rate of 10 °C/min. To prepare the TGA samples, a total of 1 L of AuNP@CMD^5%^ and AuNP@CMD^1%^ colloidal suspensions were synthesized. After centrifugation at 12,000× *g* for 15 min, the precipitates were collected and re-suspended in 20 mL of ultrapure water. The resulting dispersions were then freeze-dried to obtain AuNP@CMD powders.

### 2.5. Preparation of Sensor Fibers and AuNP@DP Conjugate for FONLISA

**Figure 1:**
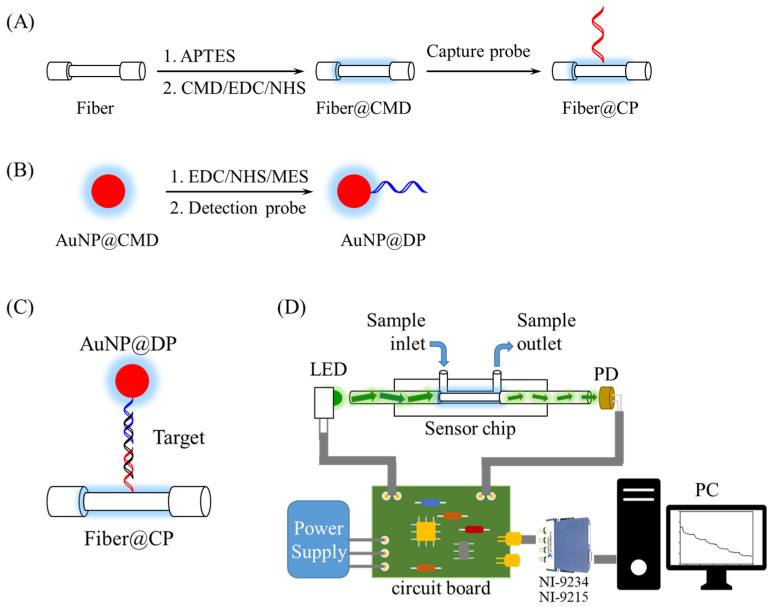
Preparation processes of FONLISA showing (**A**) the conjugation of CP on the fiber core surface via an EDC/NHS coupling reaction; (**B**) the conjugation of DP on suspended AuNP@CMD through an EDC/NHS reaction; (**C**) FONLISA detection strategy; and (**D**) schematic illustration of the experimental setup of the biosensing system.

The preparation of sensor fibers and AuNP@DP conjugate for FONLISA via conjugation of a single-stranded DNA (ssDNA) probe by EDC/NHS coupling followed the procedures similar to a previous report [2,31]. The sensor fibers functionalized with a ssDNA capture probe (CP), as shown in Figure 1A, were prepared stepwise, as follows. The bare fibers after cleaning were immersed in 1% APTES ethanolic solution for 30 min to undergo silanization, followed by being dried in an oven at 110 °C for 30 min after washing with ethanol and pure water sequentially. Secondly, a mixture of CMD (40 kDa)/EDC/NHS (0.25 mM/25 mM/50 mM) in MES buffer (0.5 M, pH 6.0) was freshly prepared and allowed to react for 30 min to enable amide-bond coupling of CMD on the fiber core surface. A CP solution (5 × 10^−7^ M) in 10 mM phosphate buffer (pH 7.4) was added to react with the CMD-modified fibers at 4 °C overnight to form a CP-functionalized fiber (fiber@CP). Then, the excess activated sites on the CMD layer were deactivated by reacting with 50 mM Tris (pH 8.3) for 30 min. Finally, the sensor fibers were washed with 10 mM phosphate buffer (pH 7.4).

To prepare AuNP@DP conjugate as shown in Figure 1B, 5 mL of a AuNP@CMD solution was centrifuged to remove excess CMD and reconstituted in 4 mL ultrapure water. It was then gently mixed with 0.2% Tween 20 for 1 h to achieve a uniform dispersion of AuNP@CMD and to prevent AuNP aggregation. Subsequently, a 1 mL mixture containing 8.7 mM NHS and 3.23 mM EDC in 0.5 M MES buffer was prepared. Afterward, 500 μL of EDC/NHS mixture and 500 μL MES buffer (0.5 M) were sequentially added to the above AuNP solution, followed by a reaction for 15 min to activate the carboxyl groups. The AuNP solution was centrifuged for 10 min at 12,000× *g*, and the resulting pellet was re-dispersed in ultrapure water. This activated solution was then reacted with a ssDNA detection probe (DP) solution at a concentration of 10^−6^ M for 16 h at 13 °C. After centrifugation (10 min at 12,000× *g*) and resuspension in Tris buffer (5 mM, pH 8.3) for 30 min to deactivate the unreacted NHS groups, a AuNP@DP solution was obtained, and its absorbance was measured by a UV−vis spectrophotometer (JASCO V-570, Easton, MD, USA).

### 2.6. Storability Test of AuNP@CMD

A AuNP@CMD solution was centrifuged at 12,000× *g* for 15 min, and the resulting pellet was redispersed in Tris buffer (5 mM, pH 8.1) containing 0.25% Tween 20. Then, the absorbance of the reconstituted solution was adjusted to 1.5 a.u., and 80 μL of the suspension was dropped onto a pre-cleaned glass fiber conjugate pad (G041, Merck, Darmstadt, Germany). The pad was then dried in a desiccator for 16 h. After drying and storing for a certain period of time, the conjugate pad was placed in a centrifuge tube, followed by the addition of 1 mL of ultrapure water. The tube was gently shaken for 10 s to release the AuNP@CMD, and the UV–vis spectrum of the resulting solution was subsequently measured.

### 2.7. Preparation and Detection of Target ssDNA Standards

A stock standard solution of target ssDNA at a concentration of 10^−6^ M was prepared in phosphate buffer (10 mM, pH 7.4) and stored at −20 °C until use. To construct a calibration curve for ssDNA detection, the stock solution was serially diluted with phosphate buffer to make standard solutions. For FONLISA, ssDNA concentrations ranging from 2 × 10^−14^ M to 2 × 10^−9^ M were used. Each 200 μL standard solution was mixed with 200 μL of AuNP@DP solution (0.45 a.u., ~0.97 nM) and incubated for 15 min, resulting in target ssDNA standards with a final concentration ranging from 1 × 10^−14^ M to 1 × 10^−9^ M. These solutions were then injected into a sensor chip sequentially, from low to high concentrations. During the injection, a target ssDNA molecule (T) will hybridize with an immobilized CP and a AuNP@DP to form a AuNP@DP–T–fiber@CP nanocomplex on the fiber core surface as shown in Figure 1C. Each injection lasted 15 min. Since the signal change is proportional to the surface concentration of the nanocomplex on the fiber core surface, the sensor readout will exhibit a molecular binding kinetic curve due to the molecular binding kinetics, and finally reach the steady-state signal when the binding reaction is at equilibrium [32], which was used to calculate the sensor response. Here, the sensor response is defined as a normalized signal ratio ΔI/I_0_ = (I_0_ − I)/I_0_, where I_0_ and I represent the steady-state signal when the sensor chip was filled with a blank and a sample, respectively. A plot of sensor response versus the logarithm of ssDNA concentration was used to establish the standard calibration curve.

### 2.8. Biosensing System and Sensor Chips

The biosensing system, as illustrated in Figure 1, was designed and constructed in accordance with previous publications [2,26,28]. In brief, it was made up of a light-emitting diode (LED, 530 nm, model IF-E93, Industrial Fiber Optic, Inc., Vernon, CT, USA), a sensor module, a photodiode (S1336-18BK, Hamamatsu, Hamamatsu City, Japan), a self-developed circuit broad integrating a LED driver circuit with 1-kHz frequency modulation and a photoreceiver amplification circuit to receive the output from the PD, a power supply (PMT-D1V100W1AA, Delta Electronics, Taipei, Taiwan), a signal acquisition module (NI-9234/NI-9215, National Instruments), and a self-developed LabVIEW-based (National Instruments, Austin, TX, USA) graphical user interface. The sensor module included a sensor chip, a chip holder to load the sensor chip, and a sample injection loop (Rheodyne 7725i, Bensheim, Germany) to load a sample into the sensor chip.

The sensor chips accommodating a sensor fiber were made in accordance with previous publications [27,29]. The bare sensor fibers were supplied by Instant NanoBiosensors Co., Ltd. (Taipei, Taiwan) with the following specifications: 7 cm optical fibers with a 2 cm partially unclad segment in the middle, core diameter = 400 (±8) μm, and buffer coating diameter = 730 (±30) μm.

## 3. Results and Discussion

### 3.1. Preparation and Characterization of AuNP@CMD

Dextran exhibits excellent biocompatibility and has been used to reduce HAuCl_4_ to AuNPs [17]. To enable bioprobe functionalization via dextran, oxidized dextran was often employed to modify a surface for subsequent bioconjugation [33,34,35]. Alternatively, carboxymethyl groups were introduced onto dextran, providing COOH moieties that can be activated by EDC/NHS chemistry for subsequent amide bond coupling with NH_2_-containing bioprobes [27,34,36]. In this study, we synthesized CMD by two different routes and applied our synthesized CMD to prepare AuNP@CMD.

**Figure 2:**
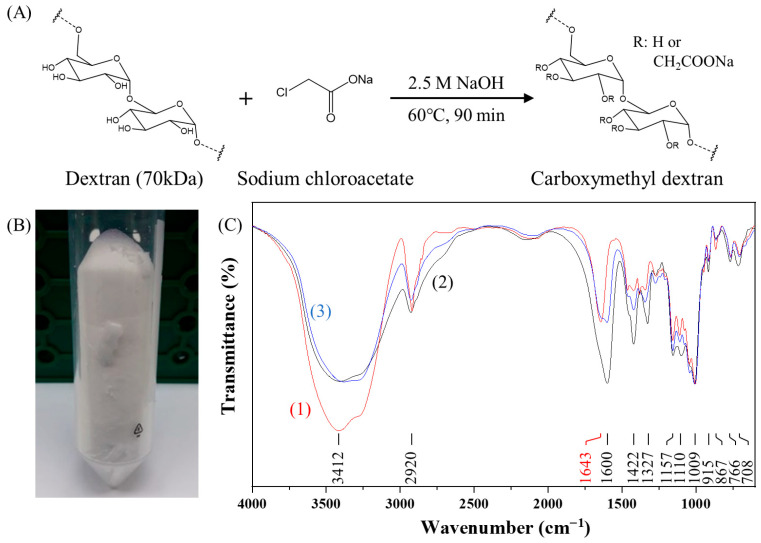
Synthesis and characterization of carboxymethyl dextran powder (CMD^P^): (**A**) Schematic of CMD^P^ synthesis; (**B**) photograph of CMD^P^; (**C**) FT-IR spectra of (1) dextran (70 kDa) (red line), (2) commercial CMD (40 kDa) (black line), and (3) synthesized CMD^P^ (70 kDa) (blue line). For comparison, the transmittances for all spectra are normalized to have the same value at 1009 cm^−1^.

As shown in Figure 2A, CMD^P^ was synthesized following the procedure described by Heinze et al. [30], in which bromoacetic acid or sodium chloroacetate can be employed under alkaline conditions via Williamson ether synthesis [23]. In this study, partial carboxymethylation of dextran was achieved by reacting dextran with sodium chloroacetate in 2.5 M NaOH at 60 °C for 90 min in a water bath. The unreacted sodium chloroacetate was removed by dialysis with a 6–8 kDa MWCO membrane, and the product was subsequently freeze-dried to yield white CMD powder (CMD^P^), as shown in Figure 2B.

To verify the partial carboxymethylation of dextran in our CMD^P^, FT-IR spectroscopy was performed (Figure 2C). The characteristic peaks at 1009 cm^−1^ and 1157 cm^−1^ correspond to the α-1,6-glycosidic bond [37,38] and the alcoholic hydroxyl (C–O) [39] of dextran, respectively. The peak at 1643 cm^−1^ is ascribed to the bending vibration of bound water with –OH groups in CMD [38,40]. The broad band in the region of 3000–3700 cm^−1^ is attributed to the combined band of O–H stretching of dextran and bound water present in dextran [38,40]. In contrast, CMD^P^ exhibited new peaks at 1600 cm^−1^ and 1422 cm^−1^, which are attributed to the asymmetric stretching and symmetric stretching of carboxylate groups (–COO^−^) [41,42], indicating that the carboxymethylation process is successful. The intensity of the 1600 cm^−1^ peak in our synthesized CMD^P^ was lower than that of commercial CMD, suggesting a lower degree of carboxymethyl substitution in our CMD^P^.

**Figure 3:**
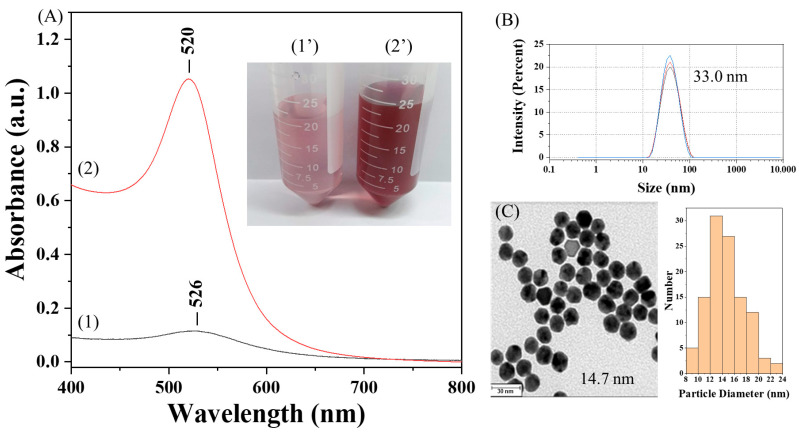
Characterization of AuNP@CMD^P^: (**A**) UV–vis absorption spectra and corresponding photographs of AuNP solutions prepared with commercial CMD (curve 1, photograph 1′) and synthesized CMD^P^ (curve 2, photograph 2′); (**B**) hydrodynamic diameter of AuNP@CMD^P^ determined by DLS; (**C**) TEM image of AuNP@CMD^P^ and corresponding size distribution (scale bar = 30 nm).

Commercial CMD showed insufficient reducing capacity, even under alkaline conditions, as evidenced by the extremely weak absorbance of the AuNP solution at the localized surface plasmon resonance (LSPR) peak wavelength (λ_p_) of ~526 nm (Figure 3A), likely due to its lower hydroxyl content as indicated by the FT-IR spectrum at 1643 cm^−1^ (Figure 2C). It has been suggested that hydroxyl groups of polysaccharides act as the reducing species for the reduction of Au^3+^ ions into Au^0^ and themselves are oxidized to carboxylic acid [18]. In contrast, our CMD^P^ preserves the reducing capability, as confirmed by the strong absorbance of the AuNP solution at λ_p_ of ~520 nm (Figure 3A). This is attributed to the retained hydroxyl groups in CMD^P^ owing to incomplete carboxymethylation. The average hydrodynamic diameter (D_h_) of AuNP@CMD^P^ was determined to be 33.0 nm by DLS (Figure 3B), while TEM analysis revealed a mean core diameter (D_c_) of 14.7 nm (n = 100) (Figure 3C).

Furthermore, the introduced carboxyl groups on AuNP@CMD^P^ could be activated by EDC/NHS chemistry and subsequently conjugated with NH_2_-containing bioprobes, enabling facile functionalization for biosensing applications.

**Figure 4:**
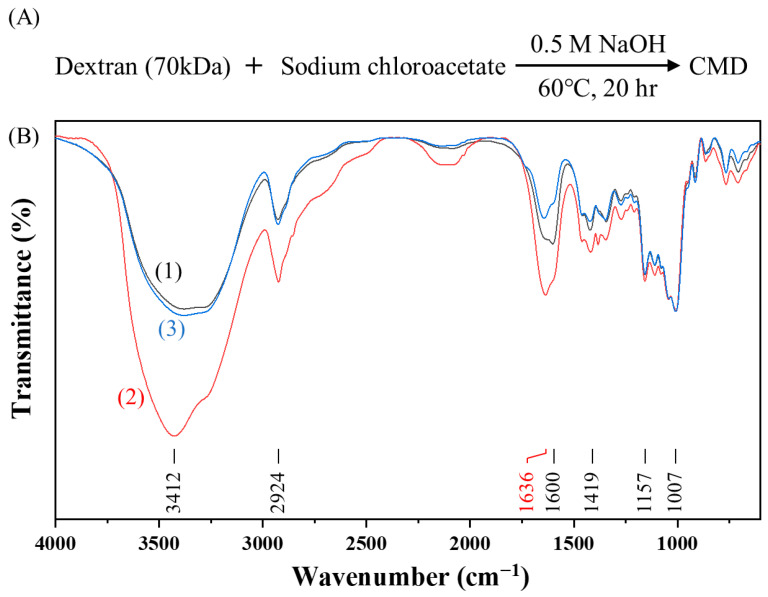
One-step synthesis and characterization of carboxymethyl dextran (CMD): (**A**) Synthetic scheme of CMD; (**B**) FT-IR spectra of (1) CMD^P^ (black line), (2) CMD^5%^ (red line), and (3) CMD^1%^ (blue line). For comparison, the transmittances for all spectra are normalized to have the same value at 1007 cm^−1^.

However, CMD^P^ is highly hygroscopic, leading to poor reproducibility in the synthesis of AuNP@CMD^P^. To overcome this limitation, we omitted the dialysis and freeze-drying steps and developed a one-step synthesis method for AuNP@CMD. In this approach, CMD solutions were prepared by lowering the NaOH concentration while extending the reaction time (Figure 4A). The resulting CMD solutions could be directly used for the synthesis of AuNP@CMD and remained stable for approximately one to two weeks at room temperature, or up to six months under frozen storage.

**Figure 5:**
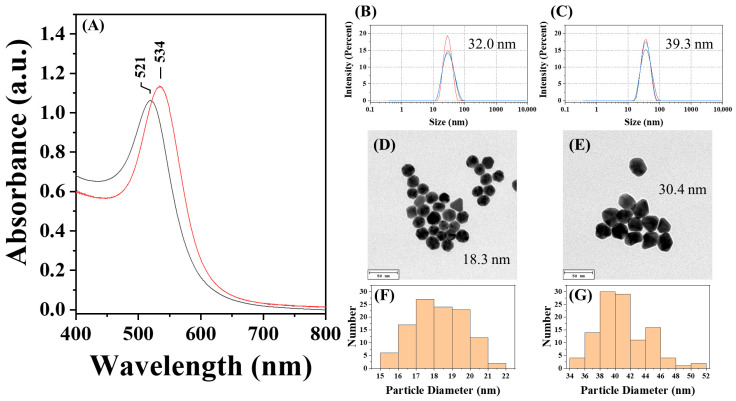
Characterization of AuNP@CMD^5%^ and AuNP@CMD^1%^: (**A**) UV–vis absorption spectra of AuNP solutions synthesized via CMD^5%^ (black line) and CMD^1%^ (red line); hydrodynamic diameter of (**B**) AuNP@CMD^5%^ and (**C**) AuNP@CMD^1%^ measured by DLS; TEM images and corresponding particle size distributions of (**D**,**F**) AuNP@CMD^5%^ and (**E**,**G**) AuNP@CMD^1%^ (scale bar = 50 nm) (n = 100).

To explore the one-step approach, two CMD solutions with two different initial dextran concentrations were prepared, and their quality as well as the presence of carboxymethyl groups were investigated by FT-IR spectroscopy (Figure 4B). As shown in Figure 5, AuNP@CMD synthesized from CMD^5%^ and CMD^1%^ solutions exhibited absorption peaks at 521 nm and 534 nm, respectively. Regarding the sizes of AuNP@CMD^5%^ and AuNP@CMD^1%^, the average hydrodynamic diameters (D_h_) were 32.0 nm and 39.3 nm, respectively, as determined by DLS, while TEM analysis revealed mean core diameters (D_c_) of 18.3 nm and 30.4 nm (*n* = 100), respectively. Stability studies further demonstrated that AuNP@CMD suspensions retained colloidal stability and reproducibility for at least one month under refrigerated storage conditions.

### 3.2. Estimation of Surface Coverage of CMD on AuNP@CMD

**Figure 6:**
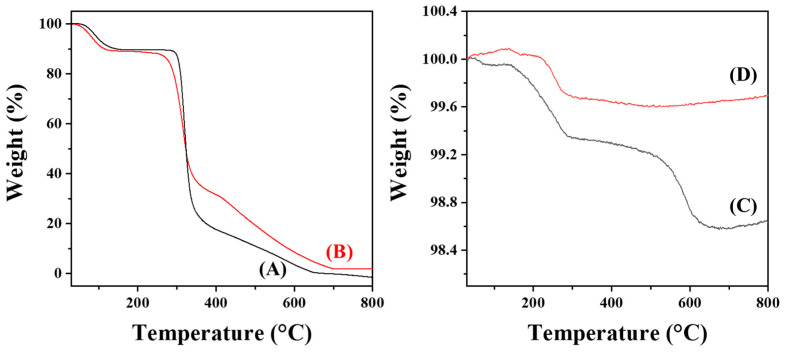
TGA curves of (**A**) dextran (70 kDa), (**B**) CMD^P^, (**C**) AuNP@CMD^5%^, and (**D**) AuNP@CMD^1%^.

The thermal stability and the average amount of CMD coated on each AuNP@CMD were investigated by thermogravimetric analysis (TGA). As shown in Figure 6A,B, a pronounced weight loss was observed at approximately 300 °C, corresponding to the thermal decomposition range of polysaccharide backbones in pristine dextran and our synthesized CMD powder (CMD^P^). However, the extent of weight loss differed between dextran and CMD^P^ [43,44]. This variation may be attributed to several factors: (1) Altered decomposition pathway: The presence of carboxymethyl groups may modify the overall distribution of thermal degradable products, promoting carbonization (formation of less volatile residues), or altering the decomposition profile. (2) Sodium salt form: Carboxymethyl dextran often exists as a sodium salt (–COO^−^Na^+^). Alkaline salts or metal cations can influence thermal degradation; certain metal salts may catalyze decomposition or enhance carbonization, leading to different residue amounts and weight-loss behaviors. (3) Degree of substitution (DS) and molecular weight distribution: Variations in DS significantly affect thermal stability. A higher DS may increase the participation of side chains in decomposition or promote cross-linking, thereby altering the percentage and temperature range of weight loss. Here, both dextran and CMD^P^ exhibited a major weight loss between 300 and 350 °C, which corresponds to the thermal degradation of the polysaccharide backbone. A subsequent gradual mass decrease occurred from 350 to 650 °C, attributed to further oxidation and decomposition of residual carbonaceous fragments. Complete combustion was achieved above 650 °C, leaving no residual mass (~0%) for dextran, while leaving a small residual mass (1.88 wt%) for CMD^P^, indicating the organic nature of both dextran and CMD^P^.

In contrast, AuNP@CMD^5%^ displayed two distinct stages of weight loss as shown in Figure 6C. The first major weight loss occurred between 140 and 290 °C, suggesting the decomposition of weakly bound organic species or CMD fragments adsorbed on the gold surface. A secondary weight loss was observed from 500 to 650 °C, corresponding to the gradual decomposition of more strongly bound or cross-linked organic residues. Above 650 °C, the curve reached a plateau, leaving a residual mass of 98.64%, which can be ascribed to the remaining metallic gold. This result indicates that approximately 1.36 wt% of the sample consists of organic matter.

For AuNP@CMD^1%^, a single major decomposition step was observed within 220–290 °C, and no significant mass loss was detected above 300 °C, as shown in Figure 6D, suggesting that the CMD layer was thinner and thermally more labile compared with that of AuNP@CMD^5%^. The final residual mass was 99.69%, corresponding to only 0.31 wt% organic content. The smaller amount of organic material indicates a lower CMD coating density.

The downward shift of the decomposition temperature in both AuNP@CMD samples compared to pure CMD suggests that the presence of gold nanoparticles alters the thermal degradation behavior of CMD. This phenomenon may be attributed to the catalytic effect of the gold surface, which facilitates the oxidative decomposition of CMD.

The average surface coverages of CMD on AuNP@CMD^5%^ and AuNP@CMD^1%^ were estimated based on the TGA and TEM data following the calculation method described by Bajaj et al. [45] and assuming the density of gold in the AuNPs to be 19.32 g/cm^3^. For AuNP@CMD^5%^, the average surface area and the average mass of a single AuNP are calculated to be 1052 nm^2^ and 6.20 × 10^−17^ g, respectively. Based on the initial sample weight of 0.01468 g and the residual sample weight of 0.01449 g, and assuming the molecular masses of CMD and pristine dextran are similar, the CMD-to-AuNP molar ratio was calculated to be 6.86. Similarly, for AuNP@CMD^1%^, the average surface area and the average mass of a single AuNP are calculated to be 2903 nm^2^ and 2.84 × 10^−16^ g, respectively. Based on the initial sample weight of 0.01942 g and the residual sample weight of 0.01935 g, the CMD-to-AuNP molar ratio was calculated to be 8.35. Further assuming that our CMD possesses a similar hydrodynamic radius as the pristine 70 kDa dextran, which is reported to have a Stokes radius of 6.39 nm [46], the average surface coverages of CMD on AuNP@CMD were estimated to be 83.7% for AuNP@CMD^5%^ and 36.9% for AuNP@CMD^1%^. These results indicate that the CMD coating on AuNP@CMD^5%^ provides a more complete and denser surface coverage compared to AuNP@CMD^1%^. It should be noted that although the calculated surface coverages are less than 100%, it has been reported that dextrans are linear, flexible, and deformable molecules [47], their actual surface coverages on AuNP@CMD may be higher than expected since their interactions with AuNP surface may allow them to deform from the roughly spherical shape to a more slab-like shape. The stability of these nanoparticles in aqueous media suggests that the AuNP core is well protected by the CMD coating, likely due to the high surface coverage of CMD on AuNP@CMD.

### 3.3. Stability and Storability of AuNP@CMD

**Figure 7:**
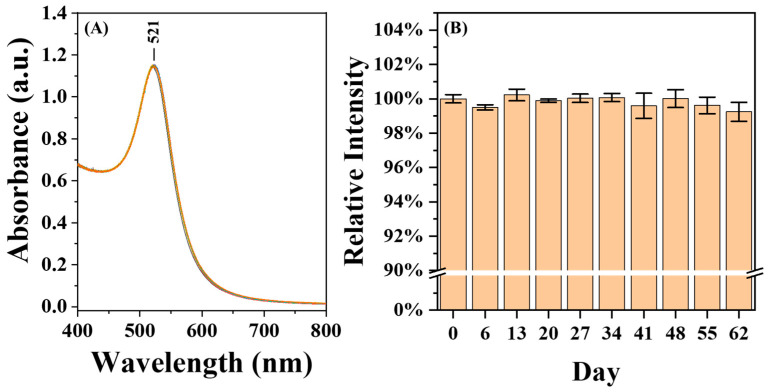
Stability and Storability of AuNP@CMD. (**A**) UV–vis absorption spectra of a AuNP@CMD solution (n = 10) monitored over a period of two months. (**B**) Relative absorbance intensities of AuNP@CMD, with the initial value (day 0) normalized to 100% for comparison.

The AuNP@CMD exhibited excellent stability. As shown in Figure 7, the UV–vis absorption spectra of the AuNP@CMD solution stored at 4 °C for two months showed less than 1% variation in absorbance intensity.

**Figure 8:**
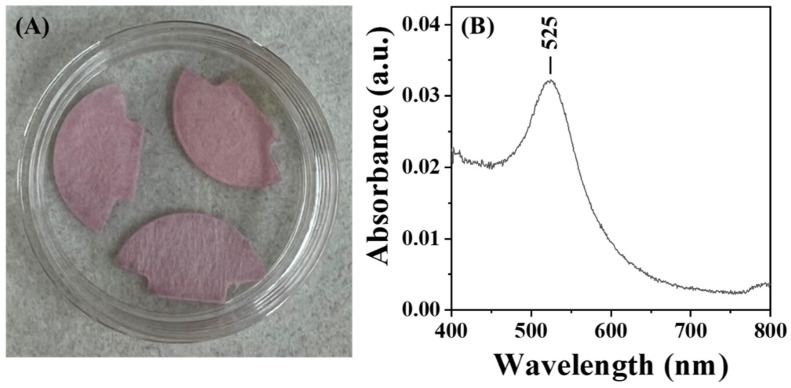
(**A**) Photograph of AuNP@CMD dried on a glass fiber conjugate pad. (**B**) UV–vis absorption spectrum of AuNP@CMD re-dispersed from the pad using ultrapure water.

For practical applications, the long-term storability of AuNP@CMD in a dried state is critical for the commercialization of POCT biosensors. To test for the storability of AuNP@CMD in the dried state, we mimicked the storage conditions often employed in the lateral flow assays. As shown in Figure 8, after being dried on a glass fiber conjugate pad, the AuNP@CMD could be re-dispersed, as evidenced by a slight shift in λ_p_ from 521 nm to 525 nm, indicating that the nanomaterial AuNP@CMD is suitable for POCT applications in plasmonic nanoparticle-linked sorbent assays.

### 3.4. Application of AuNP@CMD to Plasmonic Nanoparticle-Linked Sorbent Assay

The CMD coating on AuNPs provides steric stabilization, an antifouling surface, and available carboxyl groups for functionalization with bioprobes [36,48]. To demonstrate its applicability to plasmonic nanoparticle-linked sorbent assays, we chose our recently developed ultrahigh-sensitivity FONLISA method as a model. In this analytical performance study, a single-stranded DNA (ssDNA) with the sequence corresponding to the codon 26 region of β-thalassemia was taken as a model analyte. For clinical diagnostics of β-thalassemia, the detection of single-nucleotide polymorphisms (SNPs) in the codon 26 region is often employed. Since specific detection of SNP requires an additional biorecognition mechanism [29,49,50], this study only aims to examine the analytical performance of using AuNP@CMP as the nanoplasmonic nanoprobe in FONLISA. To minimize the effect of secondary structures in the DP and CP probes on the biosensor sensitivity assessment, as well as maintaining a reasonable DNA hybridization affinity [51], only 25 mer in the sequence corresponding to the codon 26 region was selected. Detailed procedures for the modification of a detection probe on gold nanoparticles and a capture probe on the optical fiber core are illustrated in Figure 1.

Since background nonspecific adsorption (BNA) of AuNP@DP onto the fiber core can produce false-positive signals, BNA analysis was performed to establish a cutoff value (B) to exclude sensor responses arising from BNA. For the BNA test, a AuNP@DP solution without target ssDNA was injected into a CP-functionalized sensor chip. The cutoff value B was defined as the mean sensor response due to BNA plus three times its standard deviation. Therefore, only sensor responses exceeding B were considered valid. This B value was also used to calculate the limit of detection (LOD), where the sensor response at B represents the minimum distinguishable analytical signal.

**Figure 9:**
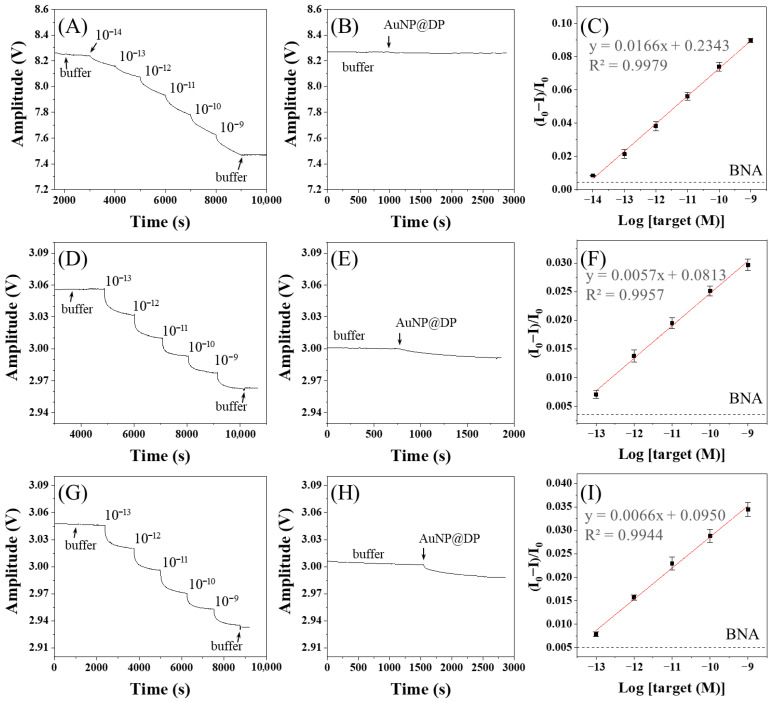
Real-time detection and calibration curves of ssDNA using FONLISA with different AuNP@CMD probes: (**A**–**C**) AuNP@CMD^P^; (**D**–**F**) AuNP@CMD^5%^; (**G**–**I**) AuNP@CMD^1%^. Sensorgrams (**A**,**D**,**G**) were recorded by sequential injection of ssDNA standard solutions in the presence of the corresponding AuNP@CMD probe. Sensorgrams of BNA tests (**B**,**E**,**H**) were recorded by injection of the corresponding AuNP@CMD probe only. (**C**,**F**,**I**) are the corresponding calibration curves. Each data point represents the mean of three independent measurements. The black dashed line indicates the B value determined from the corresponding BNA test.

The real-time sensorgrams in the detection of target ssDNA and BNA analysis using AuNP@CMD^P^ are shown in Figure 9A and Figure 9B, respectively. The sensor exhibited a wide linear response range from 1 × 10^−14^ M to 1 × 10^−9^ M (r^2^ = 0.9979). The corresponding calibration curve of the normalized response (ΔI/I_0_) versus the logarithm of ssDNA concentration (Log[ssDNA]) yields the linear regression equation y = 0.0166x + 0.2343 (Figure 9C). The B value was 0.00451, resulting in a calculated analytical sensitivity (S) and LOD of 0.0166 M^−1^ and 7.7 fM, respectively. Similarly, AuNP@CMD^5%^ was also used for target ssDNA detection (Figure 9D) and BNA analysis (Figure 9E), showing a linear range from 1 × 10^−13^ M to 1 × 10^−9^ M (r^2^ = 0.9957). The corresponding calibration curve (Figure 9F) gives the linear regression equation y = 0.0057x + 0.0813, with a B value of 0.00434, an S value of 0.0057 M^−1^, and an LOD of 25.5 fM. Detection of target ssDNA and BNA analysis using AuNP@CMD^1%^ is shown in Figure 9G and Figure 9H, respectively. The calibration curve (Figure 9I), with a linear range of 1 × 10^−13^ M to 1 × 10^−9^ M (r^2^ = 0.9944), produces a linear regression equation y = 0.0066x + 0.0950, with a B value of 0.00504, an S value of 0.0066 M^−1^, and an LOD of 28.5 fM.

Table 1 compares the UV–vis absorption spectra, particle sizes, S values, and LODs of the three types of AuNP@CMD. The particle sizes (D_h_ and D_c_) increase in the order of AuNP@CMD^P^ < AuNP@CMD^5%^ < AuNP@CMD^1%^. We attribute this phenomenon to the weakest reducing power of CMD^P^ due to the highest degree of carboxymethylation, as revealed by the relatively strong absorption at 1600 cm^−1^ in the IR spectrum. In addition, AuNP@CMD^5%^ has the narrowest size distribution as revealed by the smallest full-width at half-maximum (FWHM) in the LSPR band. We attribute this phenomenon to the high surface coverage of CMD on AuNPs to stop the further growth of the AuNPs in the supersaturated solution [52], as supported by the TGA analysis results. Finally, in comparison of the analytical performance, AuNP@CMD^P^ exhibited a higher S value and a lower LOD compared to that of AuNP@CMD^5%^ and AuNP@CMD^1%^. As shown in Figure 4B, the relative intensity of the 1600 cm^−1^ peak to the 1636 cm^−1^ peak is higher for AuNP@CMD^P^, indicating a slightly higher degree of carboxymethylation than the other two samples. The increased number of carboxymethyl groups allows for more bioprobe molecules to be immobilized, thereby enhancing the binding efficiency with an analyte. The B value of AuNP@CMD^5%^ is slightly lower than that of AuNP@CMD^1%^, likely due to a higher CMD surface coverage. However, the analytical sensitivity in principle increases with the use of a larger plasmonic nanoparticle, thereby resulting in a slightly higher S value for AuNP@CMD^1%^ than that for AuNP@CMD^5%^. Nevertheless, as compared to AuNP@CMD^5%^, AuNP@CMD^1%^ exhibits greater BNA and compromises its LOD, resulting in a higher LOD.

These results demonstrate that CMD-coated AuNPs serve as stable, reproducible, and storable plasmonic labels for application in FONLISA. The CMD coating not only prevents nanoparticle aggregation but also provides functional groups for bioconjugation, enabling ultrasensitive ssDNA detection without nucleic acid amplification. The colloidal stability of AuNP@CMD for at least one month at 4 °C further highlights CMD as an effective stabilizer for AuNPs in biosensor applications [24]. Hence, AuNP@CMD provides the desirable features to be an excellent colorimetric label for sandwich immunoassays to develop novel plasmonic nanoparticle-linked sorbent assays.

## 4. Conclusions

In this work, we developed a facile strategy to synthesize CMD-coated gold nanoparticles (AuNP@CMD) through both powder-based and one-step aqueous approaches. CMD served multiple roles by providing steric stabilization, preventing nonspecific adsorption, and introducing carboxyl groups for bioprobe functionalization. The optimized one-step synthesis enabled the preparation of AuNP@CMD with tunable particle sizes and excellent colloidal stability for at least one month at 4 °C. When integrated into the FONLISA method, the AuNP@CMD demonstrated reliable performance, achieving femtomolar sensitivity for ssDNA detection without nucleic acid amplification. These findings highlight CMD as an effective stabilizer and functional coating for AuNPs, offering a robust and reproducible nanomaterial for applications to plasmonic nanoparticle-linked sorbent assays, which are a promising approach in point-of-care diagnostics.

## Figures and Tables

**Table 1:** Characterization of various AuNP@CMD and comparison of corresponding analytical performance in FONLISA.

CMD Type	UV-Vis		DLS D_h_ (nm)	TEM D_c_ (nm)	FONLISA		
	λ_p_ (nm)	FWHM (nm)			B Value	S Value	LOD (fM)
AuNP@CMD^P^	520	82	33.0 ± 0.5	14.7 ± 1.0	0.00451	0.0166	7.7
AuNP@CMD^5%^	521	75	32.0 ± 1.2	18.3 ± 1.4	0.00434	0.0057	25.5
AuNP@CMD^1%^	532	84	39.3 ± 0.9	30.4 ± 3.6	0.00504	0.0066	28.5

## Data Availability

Data will be made available on request.

## Abbreviations

The following abbreviations are used in this manuscript:

AuNPs	gold nanoparticles
CMD	carboxymethyl dextran
AuNP@CMD	carboxymethyl dextran-coated gold nanoparticles
FONLISA	fiber optic nanogold-linked sorbent assay
ssDNA	single-stranded DNA
MES	2-morpholinoethanesulfonic acid
APTES	(3-aminopropyl) triethoxysilance
EDC	1-ethyl-3-(3-dimethylaminopropyl)carbodiimide hydrochloride
NHS	N-hydroxysuccinimide
